# Three-dimensional bioprinting sodium alginate/gelatin scaffold combined with neural stem cells and oligodendrocytes markedly promoting nerve regeneration after spinal cord injury

**DOI:** 10.1093/rb/rbac038

**Published:** 2022-06-06

**Authors:** Shuo Liu, Hui Yang, Dong Chen, Yuanyuan Xie, ChenXu Tai, Liudi Wang, Peng Wang, Bin Wang

**Affiliations:** Clinical Stem Cell Center, Nanjing Drum Tower Hospital, the Affiliated Hospital of Nanjing University Medical School, Nanjing 210000, Jiangsu Province, China; Clinical Stem Cell Center, Nanjing Drum Tower Hospital, the Affiliated Hospital of Nanjing University Medical School, Nanjing 210000, Jiangsu Province, China; Clinical Stem Cell Center, Nanjing Drum Tower Hospital, the Affiliated Hospital of Nanjing University Medical School, Nanjing 210000, Jiangsu Province, China; Clinical Stem Cell Center, Nanjing Drum Tower Hospital, the Affiliated Hospital of Nanjing University Medical School, Nanjing 210000, Jiangsu Province, China; Clinical Stem Cell Center, Nanjing Drum Tower Hospital, the Affiliated Hospital of Nanjing University Medical School, Nanjing 210000, Jiangsu Province, China; Clinical Stem Cell Center, Nanjing Drum Tower Hospital, the Affiliated Hospital of Nanjing University Medical School, Nanjing 210000, Jiangsu Province, China; State Key Laboratory of Pharmaceutical Biotechnology, Department of Sports Medicine and Adult Reconstructive Surgery, Nanjing Drum Tower Hospital, The Affiliated Hospital of Nanjing University Medical School, Nanjing 210000, Jiangsu Province, China; Clinical Stem Cell Center, Nanjing Drum Tower Hospital, the Affiliated Hospital of Nanjing University Medical School, Nanjing 210000, Jiangsu Province, China

**Keywords:** three-dimensional bioprinting, neural stem cell, oligodendrocytes, spinal cord injury

## Abstract

Accumulating research has indicated that the transplantation of combined stem cells and scaffolds is an effective method for spinal cord injury (SCI). The development of three-dimensional (3D) bioprinting technology can make the 3D scaffolds combined with cells more accurate and effective for SCI treatment. However, unmyelinated newborn nerve fibers have no nerve signaling conduction, hampering recovery of motor function. In this study, we designed and printed a type of sodium alginate/gelatin scaffold loaded with neural stem cells and oligodendrocytes, which were involved in the formation of the myelin sheaths of neural cell axons. In order to observe the effectiveness of this 3D bioprinting scaffold, we transplanted it into the completely transected rat spinal cord, and then immunofluorescence staining, hematoxylin–eosin staining and behavioral assessment were performed. The results showed that this 3D bioprinting scaffold markedly improved the hindlimb motor function and promoted nerve regeneration. These findings suggested that this novel 3D bioprinting scaffold was a good carrier for cells transplantation, thereby enhancing spinal cord repair following injury.

## Graphical Abstract

**Figure:**
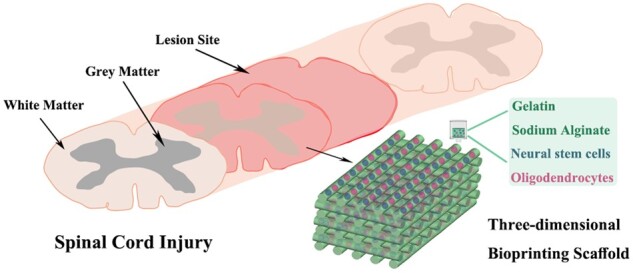

## Introduction

As a global health problem, spinal cord injury (SCI) has become one of focal points of neuroscience. Currently, although some achievements have been gained in basic science and clinical research, there are few effective therapeutics for acute or chronic SCI [1, 2]. The spinal cord occurred a series of tissue disorganization after SCI, axon demyelination, local neuronal atrophy and death, glial scar hyperplasia, chronic inflammation and nutritional deficiency [3, 4]. The inhibitory microenvironment formed by these complex pathological processes affected nerve regeneration [5]. In these circumstances, creating a suitable microenvironment and providing necessary supports were two key factors for nerve repairing [6, 7]. In recent years, three-dimensional (3D) bioprinting technology has become a powerful tool to construct artificial tissue and organ structure in tissue engineering field [8–10]. Cells, hydrogels and biomolecules were combined to generate 3D tissue models in a layer-by-layer manner in 3D bioprinting [11]. Compared with traditional scaffold manufacturing technologies, 3D bioprinting has several advantages, including accurate control of the pore size and geometry of the scaffold, uniform and reproducible manufacture, and precision location of cells [12]. Scaffolds with complex biological microstructures could be produced by 3D bioprinting technology [13]. This was an advantage for the repair of injured central nervous system (CNS) with complex structure and function.

Biomaterials for 3D bioprinting can be divided into natural and artificial materials. Alginate, as a readily and available natural biopolymer, was typically used to manufacture hydrogels due to its long-term stability and water retaining ability [14]. Alginate has the advantages of biocompatibility, degradability and non-toxicity, but the weaknesses of low bioadhesion and biological inertia limited its application in tissue engineering [15]. Gelatin is a kind of protein, which is hydrolyzed from animal origin collagen (such as pig, bovine or fish collagen), bones and connective tissues [3, 16]. The clinical application of gelatin was approved by the US Food and Drug Administration (FDA) because of its degradability, biocompatibility, non-toxicity and low antigenicity. Thus, it is widely used in the biomedical sector (like drug delivery, pharmacy, gene therapy, wound healing, regenerative medicine and tissue engineering) [17]. In addition, gelatin is a widely used biomaterial for cell culture because it retains the bioactive sequence of collagen [15, 18]. These advantages make it possible to create a microenvironment suitable for cell adhesion, proliferation, migration and differentiation [16, 19]. The combination of alginate and gelatin can not only solve the problem of cell adsorption, but also improve the mechanical strength and prolong the degradation time of the scaffold.

Researches have revealed that the reconstruction and implantation of exogenous stem cells can effectively promote the recovery of injured spinal nerve tissue [20, 21]. In the treatment of SCI, exogenous stem cells can differentiate into new glial cells and neurons to supplement the damaged neural cells. In addition, various kinds of extracellular matrix secreted by stem cells in the process of proliferation and differentiation can fill the cavity in the injured site, thus providing a framework and support for axon regeneration. Moreover, the produced nutrients can improve the regeneration state of microenvironment and promote neuronal axon regeneration [22]. Neural stem cells (NSCs) have the potential to differentiate into neurons which could replace damaged spinal cord tissue and/or secret multiple neurotrophic factors for recovery of SCI. Therefore, NSCs have been widely applied in the study of SCI as seeding cells [23–25]. Myelin stretches from glia to envelop axon segments as a particular membrane, which supports axonal function and accelerates nerve signaling conduction. Oligodendrocytes (OLGs) could extend multiple processes and form several myelinating segments simultaneously [26, 27]. OLGs and their precursor cells (OPCs), which myelinate the neuron axons and accelerate nerve signaling conduction in healthy tissue, respond to SCI by differentiating and producing myelin [28]. It has been demonstrated that transplantation of exogenous OPCs is conducive to promote regeneration and motor recovery after SCI [29, 30].

Based on studies aforesaid, we synthesized a 3D bio-printing hydrogel using sodium alginate and gelatin to construct a 3D structure with uniform pores through 3D bioprinting technology, where NSCs and OLGs were employed as seeding cells. The scaffold combined with NSCs and OLGs was transplanted into the completely transected spinal cord of rat. The results showed that this 3D bioprinting scaffold could improve myelinated nerve regeneration and markedly promote the hindlimb motor function, indicating this novel 3D bioprinting scaffold was an advantageous carrier for cells transplantation to treat SCI.

## Materials and methods

### Culture and identification of NSCs and OLGs

The isolation of NSCs could refer to our previous research [5]. Briefly, the hippocampi of rat embryos at Day 12 after pregnancy (provided by the Laboratory Animal Center of Nanjing Medical University (license No. SYXK (Su) 2014-0052)) were extracted and digested in accutase for 20 min at 37°C. After phosphate-buffered saline (PBS) rinsing, the hippocampi tissue was evenly mixed with complete Dulbecco’s modified Eagle medium/Nutrient Mixture F-12 (DMEM/F12) medium (Gibco, Grand Island, NY, USA). The medium contained penicillin–streptomycin (1%, Gibco), B27 supplement (2%, Gibco), basic fibroblast growth factor (bFGF, 20 ng/ml, Peprotech Asia, Rehovot, Israel) and epidermal growth factor (20 ng/ml, Peprotech Asia). The cell suspension was filtered and transferred to tissue culture flasks. The primary NSCs could grow into neurospheres (Passage 0, P0) in complete DMEM/F12 medium at Day 4 after isolation from embryo. Then neurospheres of NSCs were digested into unicellular cells to proliferate into new neurospheres after 3–4 days (P1). Neurospheres of NSCS at P3 were used for identification and 3D bioprinting. For identification of NSCs, neurospheres of NSCs were seeded in 12-well culture plate (Corning, NY, USA) and the adhered neurospheres were performed immunostaining against Nestin antibody, a marker of NSCs. To further identifying the neuronal differentiation potentials of NSCs, neurospheres were digested into unicellular cells, which were seeded at 3 × 10^5^ cells per well in 12-well culture plate (Corning, NY, USA) in differentiation medium (DMEM/F12 containing 2% B27). After 4–5 days in differentiation medium, the differentiated cells were assessed by immunostaining against anti-glial fibrillary acidic protein (GFAP, a marker of astrocytes), anti-beta III tubulin (Tuj-1, a marker of neurons), anti-myelin basic protein (MBP, a marker of mature OLGs) and anti-sex determining region Y-box 10 (SOX10, a marker of OLGs) antibodies.

OLGs were cultured by shaking off method [31, 32]. Tissue culture flasks (Corning) coated with poly-l-lysine (Sigma-Aldrich, USA) were used in the culture of OLGs. Commonly, mixed glial cells cultures were obtained from the cerebra of newborn rats and cultured in DMEM medium containing 15% fetal bovine serum (Gibco). The medium was changed every 3 days. After 10 days, the flasks were shaken for 2 h at 160 rpm, and the medium was changed followed by another 16 h at 200 rpm at 37°C. These operations were to separate the OPCs grown on the top of the confluent layer of astrocytes. The cell suspension was transferred to a new petri dish for 2 h. The purpose of this step was to remove microglia and astrocytes. The supernatant of OPCs was collected and centrifuged for 6 min at 1000 rpm. The cells precipitation was mixed with DMEM/F12 medium containing 2% B27, platelet-derived growth factor-AA (PDGF-AA, PeproTech) and bFGF. Then the cells were inoculated in Petri dishes coated with poly-l-lysine with 2 × 10^5^ cells/cm^2^. After OPCs were cultured to the third generation, it was induced to differentiate into OLGs by adding 30 nM 3,5,3′-triiodothyronine (T3, Sigma-Aldrich, USA).

CellTracker CM-Dil (a living cell marker, Invitrogen, Carlsbad, CA, USA) was used to label NSCs and OLGs. In this way, we could track the transplanted NSCs and OLGs *in vivo.* The cell precipitation was mixed with CM-Dil working solution (20 μg/ml), and then incubated at 37°C for 15 min and then at 4°C for another 15 min. The CM-Dil working solution was removed by centrifugation and then the labeled cells were washed twice with PBS for bioprinting.

### Assembly of 3D printed scaffold construct

The suitable concentration of hydrogels biomaterial is generally 10–20% [33]. In this study, the ratio of sodium alginate/gelatin was 2.5%:7.5%, eventually forming a 10% hydrogels material. This concentration of sodium alginate and gelatin gave a suitable viscosity for 3D bioprinting. Before printing, gelatin and sodium alginate were dissolved in PBS to prepare hydrogels. The cell suspension was gently mixed with the hydrogels to achieve a final concentration of 2.5% sodium alginate, 7.5% gelatin, and 3 × 10^6^ NSCs/ml, and 3 × 10^6^ OLGs/ml. The 3D Bio plotter (Regenovo, China) was used for building a cuboid model with grid structure. Subsequently, the hydrogels precursor was printed by mechanical dispensing system. The diameter of nozzle orifice was 200 μm. The printing speed and pressure were 1–10 mm s^−^^1^ and 20–50 psi, respectively. The temperature of the substrate was 15°C. The 3D bioprinting scaffold was generated by layer-by-layer deposition with 200 µm channel-to-channel spacing. The volume of printed scaffold was about 3 × 2 × 3 mm^3^ (w × h × l). When each scaffold printing was completed, the scaffold was crosslinked in CaCl_2_ solution (200 mM) for 6 min. The sum of NSCs and OLGs in the scaffold was about 2 × 10^4^ (NSCs (3 × 10^3^/μl) and OLGs (3 × 10^3^/μl), total volume ≈ 3.5 µl). The 3D printing scaffolds were put in dishes which were sent to the animal experiment center for transplantation in a medical incubator at 4°C. It took about 1 h from the bioprinting to the transplantation.

### Scanning electron microscope

The morphology of lyophilized hydrogels was characterized by scanning electron microscope (SEM, S-3400N, Hitachi, Japan). The hydrogels were dehydrated using ethanol solution step-by-step (30, 50, 70, 90, 95, and 100 wt %) and freeze-dried. Finally, they were sputter-coated with gold and imaged with SEM.

### Rheological measurements

The rheological properties of sodium alginate/gelatin hydrogels were measured using a rheometer (Thermo Scientific Haake Mars 40, USA) with cone-and-plate geometry (1°/20 mm, gap: 0.2 mm). Storage modulus (G′) and loss modulus (G″) were estimated by using a frequency-sweep test at a frequency of 0.01–100 rad s^−^^1^ with 1% strain or a strain amplitude range of 0.01–10% at a frequency of 6.28 rad s^−^^1^. All the rheological experiments were performed at 37°C.

### Compression tests

The Young modulus and fracture strength of fabricated hydrogels were detected using compression tests. The compressive stress–strain was assessed by using an Instron-5944 universal instrument equipped with a 2 kN sensor at room temperature. In the compression-crack tests, the rate of compression was maintained at 2 mm/min. The Young modulus was obtained by approximate linear fitting values under 35–40% strain deformation. The tests were repeated thrice.

### Cell viability

The viability of NSCs and OLGs embedded in sodium alginate/gelatin hydrogels after extrusion was investigated by staining with Calcein-AM/propidium iodide (PI) (Dojindo, Japan). The 3D bioprinting scaffolds were seeded in 12-well culture plate (Corning) with complete DMEM/F12 medium and the medium was changed every other day. Cultured for 3 and 5 days, the scaffolds were performed cell viability test at Days 3 and 5, respectively. Three-dimensional bioprinting scaffolds were washed with PBS 2 times and then were immersed in 100 μl staining solution (PI at 4.5 μM and Calcein-AM at 2 μM) for 30 min at 37°C in dark. Then, the scaffolds were washed with PBS two times. Leica DMi8 Confocal Microscope (Leica Microsystems, Wetzlar, Germany) was used to take images of samples. Each image was obtained by *z*-axis superposition. We randomly selected six visual fields of each sample (*n* = 3) at 200× magnifications for viability statistics.

Cell viability = the number of living cells (green, living cells)/(number of living cells + number of dead cells (red, living cells)) × 100%

### Surgical procedures for SCI model

All animal experiments were conducted in accordance with the Guides for the Care and Use of Laboratory Animals from the National Institutes of Health. The animal experiments were approved by the Research Ethics Board of Nanjing Drum Tower Hospital, the Affiliated Hospital of Nanjing University Medical School (approval no. 2021AE02019).

Female Sprague-Dawley rats (*n* = 30, 200–250 g, provided by the Laboratory Animal Center of Nanjing Medical University, specific pathogen free) were kept in humidity and temperature-controlled animal environment. All rats were randomly divided into the scaffold group (*n* = 10, 3D bioprinting scaffold transplant after SCI), the cells/scaffold group (*n* = 10, 3D bioprinting scaffold co-cultured with NSCs and OLGs transplant after SCI) and the SCI group without any treatment (*n* = 10). All rats were anesthetized with isoflurane (RWD, Shenzhen, China). The T7–9 vertebrae of rats were exposed by a 2-cm midline incision, and a 3-mm fragment spinal cord was completely transected at the T8–9 vertebrae. A bioprinting scaffold loaded with NSCs and OLGs was transplanted to the site of spinal cord defect, and the incision were sewn. After the animals came round from anesthesia, they were returned to the cages. Antibiotics (Yuekang, Beijing, China) were injected into the rats for 7 days after surgery. Each rat was offered routine post-operative care and micturated manually every day.

### Histological analysis

All animals were sacrificed at 8 weeks after operation. Animals were anesthetized with isoflurane, PBS and 4% paraformaldehyde were used for perfusion. The spinal cord tissue with a length of about 2–3 cm near the injury was obtained. The spinal cord tissue was fixed in 4% paraformaldehyde in PBS for 50 h at 4°C. The tissue was dehydrated using sucrose solution (20% and 30%) and embedded in optimal cutting temperature compound. Finally, the tissue samples were cut into 8-μm-thick sections on a Leica CM1900. Cellular and extracellular matrix features were observed generally by hematoxylin and eosin (H&E) staining of tissue sections. The frozen section was fixed followed by H&E staining. After water rinsing, the frozen section was dehydrated by gradient alcohol series. Xylene and neutral resin were used to clear and seal the frozen section. The primary antibodies used for immunofluorescence staining were as follows: anti-Nestin (1:500, mouse, Cat# ab6142, Abcam, Cambridge, UK), anti-Tuj-1, 1:500, mouse, Cat# ab7751, Abcam), anti-GFAP, 1:500, rabbit, Cat# ab7260, Abcam), neurofilament (NF, 1:200, mouse, Cat# ab3966, Abcam), anti-5-hydroxytryptamine (5-HT, 1:300, rabbit, Cat# 200802, Immunostar, Hudson, WI, USA), anti-MBP (1:500, rabbit, Cat# ab40390, Abcam) and anti-SOX10 (1:250, mouse, Cat# SC365692, Santa Cruz, USA) antibodies. Primary antibodies were incubated overnight at 4°C. Sections then were incubated with corresponding secondary antibodies (Alexa Fluor 488 goat anti-rabbit IgG, Cat# A11034, Alexa Fluor 488 donkey anti-mouse IgG, Cat# A21202, all 1:500, Invitrogen) for 1 h at room temperature. 4ʹ,6-Diamidino-2-phenylindole (DAPI, Cat# ab104139, Abcam) was used to stain cell nuclei. Leica DMi8 confocal microscope was used to captured images.

Image J software (National Institutes of Health, USA) was used to quantify immunostaining-positive cells at the injured sites. To estimate the density of GFAP-positive cells in the lesion center of rat spinal cord, only the astrocytes near or wrapped the DAPI stained nucleus were counted for evaluating. We randomly selected 4–6 visual fields of each section at 400× magnifications, and 8–10 sections of injured spinal cord were estimated per rat. The quantitative method of Tuj-1-positive cells, NF-positive cells, MBP-positive cells and 5-HT-positive cells was the same as that of GFAP-positive cells.

### Behavioral assessment

Basso, Beattie and Bresnahan (BBB) locomotor rating scale was performed to assess the hindlimb motor function of all rats [34]. The BBB evaluation was detected by two independent experimenter who were unaware of the treatments. The scores of hindlimb function were obtained every week after SCI. The BBB scale scores range from 0 (complete paralysis) to 21 points (normal motor function). Rats were admitted to move freely because of spontaneous hind limb movement in an open field. The assessment took 5 min.

### Statistical analysis

The significance estimates and graphs were generated by GraphPad Prism version 8.0 (GraphPad Software, San Diego, CA, USA). The data were expressed as mean ± standard derivation (SD) from a minimum. If the data were normally distributed, a one-way analysis of variance was applied followed by Tukey’s post hoc test for pairwise comparison. Each experiment was repeated three times. A *P* values < 0.05 meant statistical significance.

## Results

### Culture and identification of NSCs and OLGs

**Figure 1.**
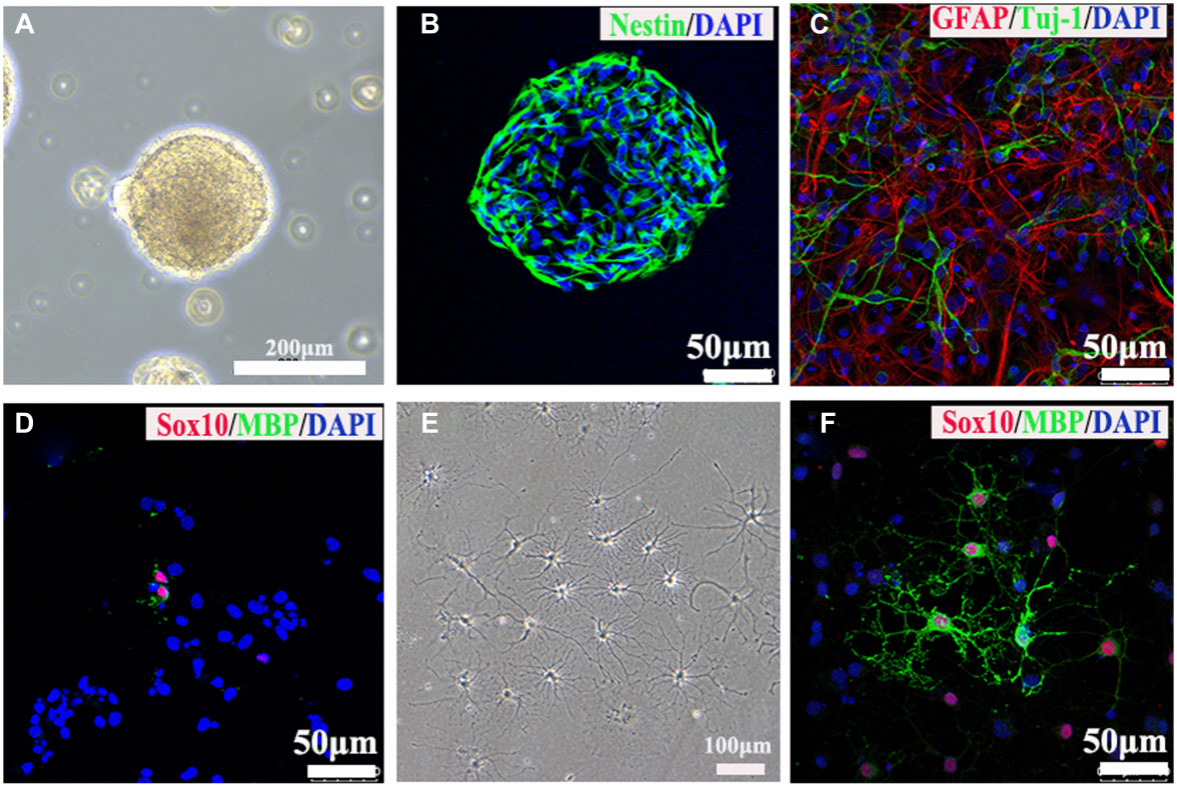
Identifications of NSCs and OLGs. (**A**) The primary cultured NSCs formed neurospheres under light microscope. (**B**) Immunostaining image showed NSCs positively expressed nestin protein. (**C**, **D**) Images of immunostaining against tuj-1 (neuron marker), GFAP (astrocyte marker), SOX10 (OLGs marker) and MBP (mature OLGs marker) antibodies. (**E**) The primary mature OLGs extended multiple processes under light microscope. (**F**) Images of immunostaining against markers of OLGs including MBP and Sox10.

Primary cultured NSCs grew into neurospheres after 4 days as shown in Fig. 1A under a light microscope and were positive for Nestin immunostaining (Fig. 1B). The immunostaining results showed that NSCs had strong differentiation potentials of astrocytes and neurons (Fig. 1C). NSCs also have the potential to differentiate into OLGs. The immunostainings of anti-MBP and SOX10 antibodies was performed to assay the differentiation of OLGs and only a small fragment of NSCs could differentiate into OLGs, consistent with previous studies [35, 36] (Fig. 1D). Thus, in this study, we cultured primary OPCs to differentiate enough OLGs for 3D-bioprinting. The differentiated mature OLGs extended multiple processes under light microscope (Fig. 1E) and positively expressed the markers of OLGs such as SOX10 and MBP (Fig. 1F).

### Characteristics of alginate/gelatin hydrogels

**Figure 2.**
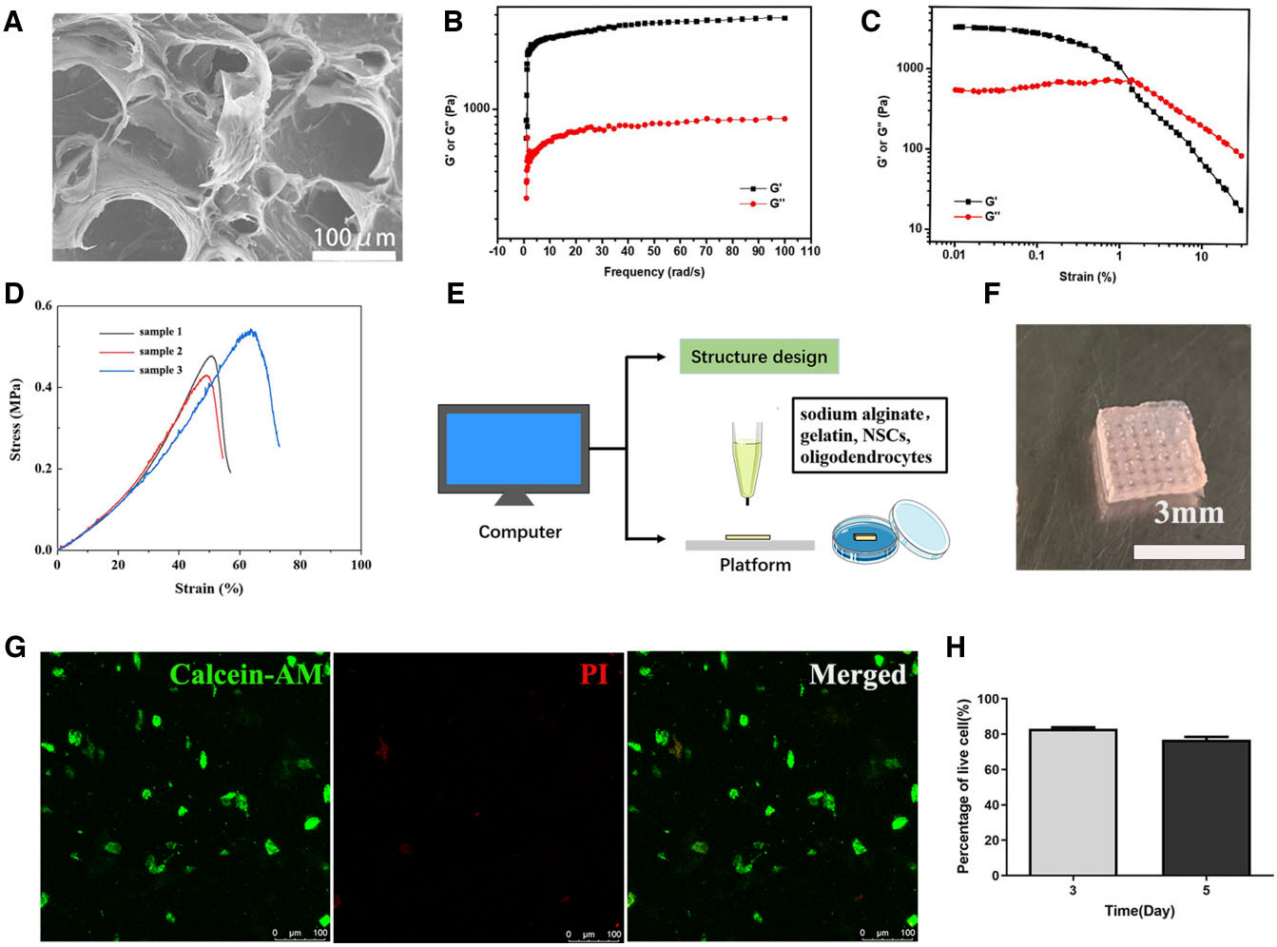
Characteristics and biocompatibility of sodium alginate/gelatin hydrogels. (**A**) SEM of porous hydrogels. (**B**) G′ and G″ of sodium alginate/gelatin hydrogels measured in frequency sweep test (from 0.01 to 100 rad s^−1^, 1% strain). (**C**) G′ and G″ of sodium alginate/gelatin hydrogels detected in a strain sweep test (from 0.01 to 10% strain, 6.28 rad s^−1^). (**D**) Compressive mechanical properties of hydrogels, uniaxial stress–strain curves under compression until cracking. (**E**) Schematic diagram of 3D bioprinting. (**F**) Sodium alginate/gelatin scaffold with regular structures was loaded with NSCs and OLGs. (**G**) Images of Calcein-AM/PI staining at Day 5. Calcein-AM indicated live cells and dead cells were labeled with PI. (**H**) The quantification of living NSCs and OLGs at Days 3 and 5.

SEM was performed to investigate the microstructure of the sodium alginate/gelatin hydrogels. According to SEM image in Fig. 2A, the porous microstructure could be observed in the hydrogels.

Furthermore, the rheological properties of the sodium alginate/gelatin hydrogels were further investigated using a rheometer and the results of the frequency-sweep (Fig. 2B) and strain-sweep (Fig. 2C) tests confirmed the great mechanical strength. Next, we quantitatively measured the compressive mechanical properties of sodium alginate/gelatin hydrogels by standard mechanical tests. The stress–strain curves from compression-crack tests of hydrogels were presented in Fig. 2D. The hydrogels could be compressed to a strain of 54.42 ± 7.98%, indicating excellent load bearing ability of the hydrogels. The Young modulus of the hydrogels was 590 ± 31 kPa.

### Biocompatibility of hydrogels

NSCs and OLGs were mixed with sodium alginate/gelatin hydrogels, followed by 3D bioprinting, then the scaffold was fixed with CaCl_2_ to obtain cells/scaffold (Fig. 2E). The scaffold had a uniform grid structure (Fig. 2F). Calcein-AM/PI staining indicated that NSCs and OLGs had good viability in the scaffold (Fig. 2G). The average cell viability was about 83% after 3 days, and it decreased to 76% after 5 days (Fig. 2H). These results indicated that 3D bioprinting sodium alginate/gelatin scaffold had a good biocompatibility and provided a suitable microenvironment for adhesion and survival of NSCs and OLGs.

### Transplantation of 3D bioprinting scaffold loading NSCs and OLGs remodeled the injured spinal cord tissue structure and locomotor functional recovery

**Figure 3.**
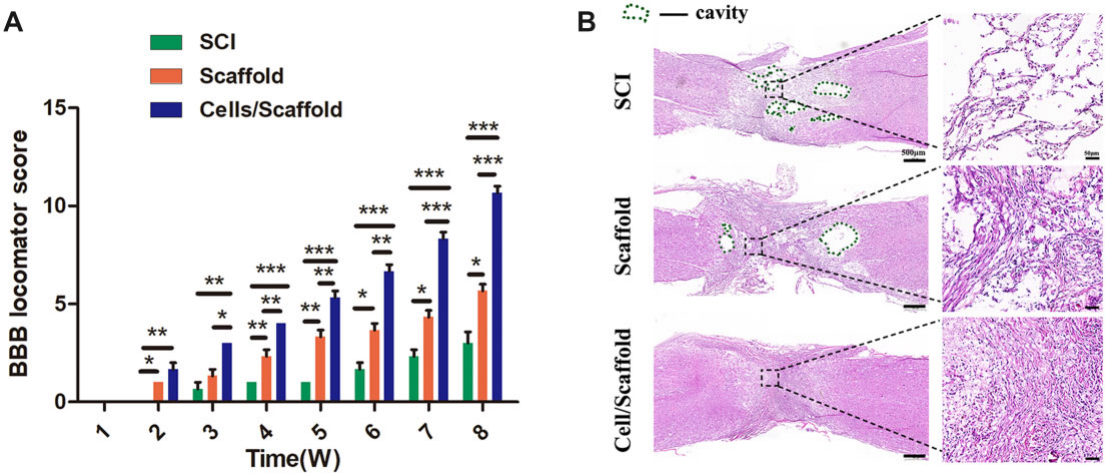
Motor functional recovery and histological evaluation. (**A**) The BBB score analysis at indicated time points. Data were expressed as mean ± SD (*n* = 10). ***P <* *0.001, **P <* *0.01, *P <* *0.05. (**B**) H&E staining of spinal cord tissues. The cavity area in the perilesional area in cells/scaffold group was significantly smaller than those in other two groups. The green dotted line indicated the cavity area.

The recovery of motor function is the most crucial index and goal in the treatment of SCI [37]. The BBB scores of all rats were 21 before operation. BBB scores were almost 0 in the first week after operation, the recovery of hindlimbs motor function was observed over time in three groups. Obviously, the SCI group had the slowest recovery speed among the three groups. The BBB scores of SCI group were only two to three at Week 8 after operation (Fig. 3A). Scaffold transplant only increased slightly BBB score, about five to six points. In contrast, the recovery of locomotor function was consistent in rats transplanted with cells/scaffolds during the observation period. The BBB scores (about 10–11 points at week 8) in cells/scaffold group were significantly higher than that in SCI and scaffold groups (*P *<* *0.001; Fig. 3A). These results indicated that the 3D bioprinting scaffold loaded with NSCs and OLGs markedly promoted the recovery of motor function after complete-transection SCI in rats.

Functional recovery was attributed to remodeling in tissue structure and pathophysiology [38]. Outcomes of H&E staining illustrated the advantages of the 3D bioprinting scaffold with NSCs and OLGs in promoting damage repair. The SCI group showed a loose and disordered structure at the site of injured spinal cord tissue, and in the cells/scaffold group, no obvious cavity was detected (Fig. 3B).

### Transplantation of 3D bioprinting scaffold loading NSCs and OLGs improved nerve regeneration in SCI rats

**Figure 4.**
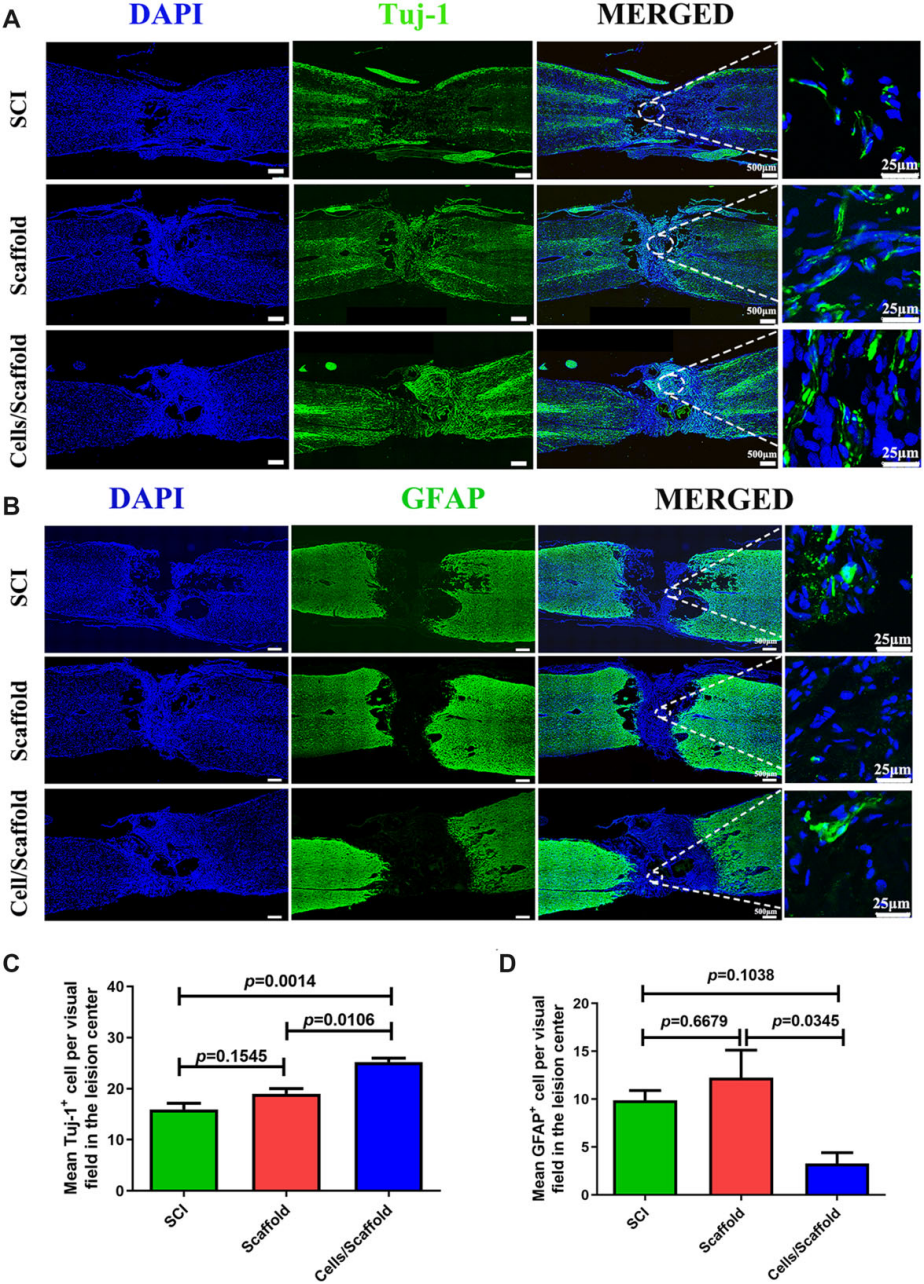
Three-dimensional bioprinting scaffold with NSCs and OLGs promoted nerve regeneration and inhibited astrocytic formation *in vivo*. (**A**, **B**) Immunofluorescence stainings for tuj-1 and GFAP. A much larger number of tuj-1-positive cells were observed in the cells/scaffold group compared with the scaffold and SCI groups. The quantity of GFAP-positive astrocytes at lesion site area in the cells/scaffold group was lower than that in the SCI and scaffold groups. (**C**, **D**) Quantification of tuj-1-positive cells and GFAP-positive cells in SCI, scaffold and cells/scaffold groups.

Recent researches suggested that newly differentiated neurons originated from exogenous or endogenous NSCs generated a relay which went through the lesion site to reconnect the two stumps after SCI [39, 40]. The staining density of Tuj-1 was assessed 8 weeks after SCI. Immunofluorescence staining of longitudinal sections showed that the expression of Tuj-1 in the cells/scaffold group was noticeably higher than that in scaffold group (*P *=* *0.0106) and SCI group (*P *=* *0.0014) (Fig. 4A and C). In contrast, a larger number of GFAP-positive cells were detected at lesion site in the scaffold and SCI groups, especially in the scaffold group. The cells/scaffold group had a significantly decrease in reactive astrogliosis (Fig. 4B and D).

**Figure 5.**
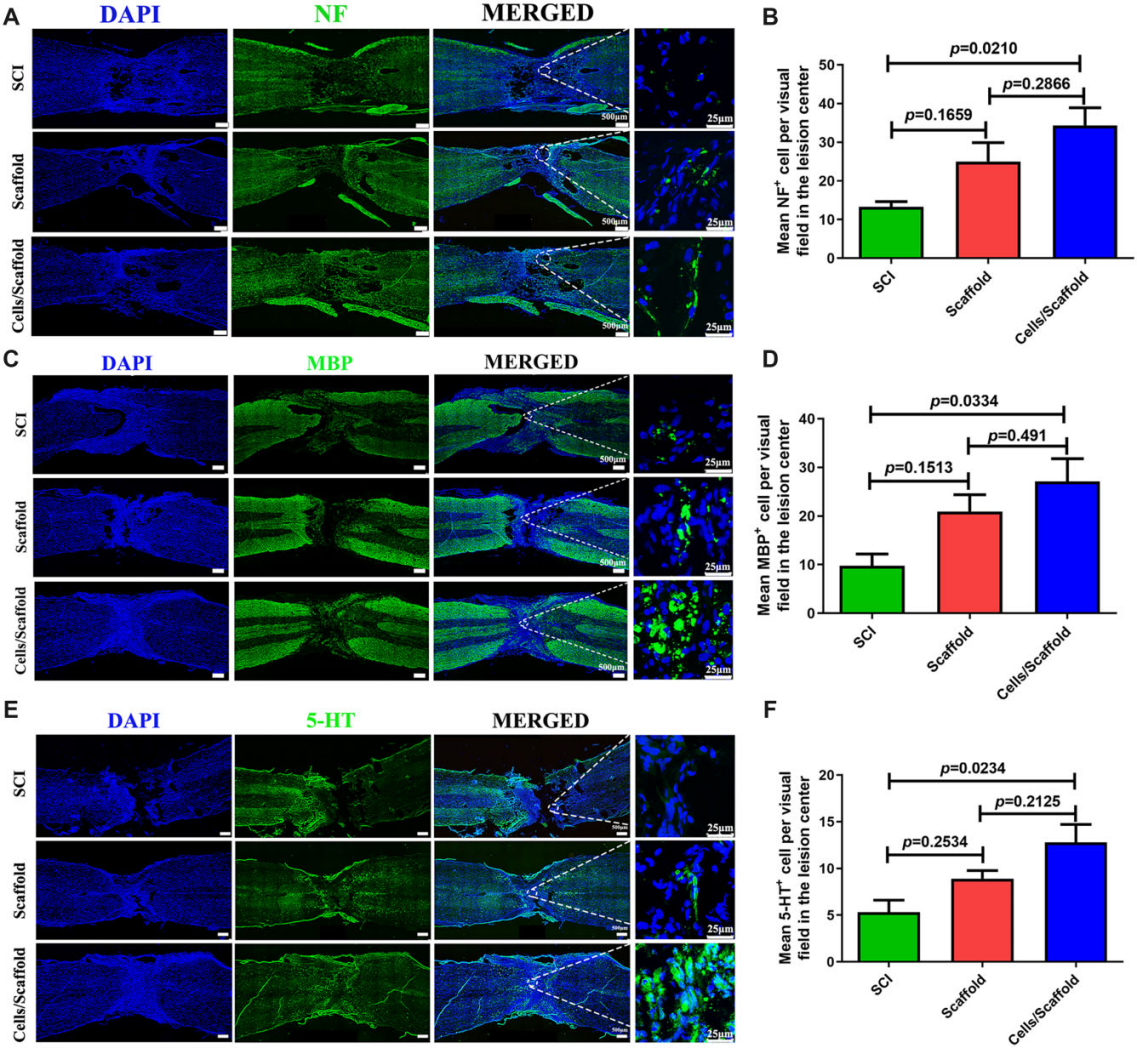
Three-dimensional bioprinting scaffold with NSCs and OLGs promoted myelinization and regeneration of axons. Immunofluorescence staining against NF (**A**), MBP (**C**) and 5-HT (**E**). A significantly higher number of axon (NF-positive cells, MBP-positive cells and 5-HT-positive cells) were observed at lesion site of SCI in cells/scaffold group compared with scaffold and SCI groups; 3D bioprinting sodium alginate/gelatin scaffold with NSCs and OLGs implantation showed successful remyelination of the regenerated nerve fibers. Quantifications of NF-positive cells (**B**), MBP-positive cells (**D**) and 5-HT-positive cells (**F**) in three groups.

Axonal regeneration is essential for the transmission of nerve signals among injured spinal cord segments. Myelin basic protein marker MBP staining and neurofilament marker NF staining were conducted to recognize the remyelination of regenerated axons at injured spinal cord. The results indicated more regenerated fibers at lesion site of rats in cells/scaffold group (Fig. 5A–D) than that in other two groups. The immunofluorescence staining result showed that NF specific nerve fibers were distributed throughout the injured site in three groups 8 weeks after operation (Fig. 5A). Quantitative analysis indicated that more NF-positive axons were regenerated in cells/scaffold group in comparison to the SCI (*P *=* *0.021) and scaffold groups (*P *=* *0.2866) (Fig. 5B). The amounts of MBP-positive axons of lesion area in cells/scaffold group were increased obviously compared with SCI (*P *=* *0.0334) (Fig. 5D). Although the number of MBP-positive cells in cells/scaffold group was more than that in scaffold group, there was no statistics difference in the two groups (*P *=* *0.491). It has been indicated that 5-HT nerve fiber is contributes to regulate the activity of spinal cord networks and participate in vertebrate locomotor behavior after SCI [41]. 5-HT immunostaining was performed to investigate whether regeneration differences existed among the three groups (Fig. 5E). The cells/scaffold group had more 5-HT-positive axons than SCI (*P *=* *0.0234) group (Fig. 5F).

## Discussion

At present, the treatment of SCI remains enormous challenges. The chief reason is that the regeneration ability of human CNS is weak [42]. Indeed, the efficacies of some clinical treatment methods such as salvage surgery, drug therapy and rehabilitation training are limited [43]. In this study, we have constructed a biological scaffold with specific structure through 3D bioprinting technology. The scaffold was printed based on sodium alginate/gelatin hydrogels and combined with NSCs and OLGs. This scaffold had a very good biocompatibility for NSCs and OLGs. This 3D bioprinting scaffold loaded with cells was transplanted into the completely transected rat spinal cord at the T8–9 level. The results indicated that compared with the scaffold and SCI groups, cells/scaffold group could significantly improve nerve regeneration and motor functional recovery. The application of 3D bioprinting technology can accurately control the structure of the scaffold. The regular network structure of scaffold was supposed conducive to the directional extension of newly neurons, and advanced regeneration of axonal, leading to increased recovery after surgery.

Scaffold material is an important factor in tissue engineering technology. The effectiveness of bio-scaffolds has been proved in delivering drugs and cells to the CNS [44]. Materials such as nanomaterials, acellular tissue matrix, and polymers have their own distinctive advantages in SCI repair. The ideal scaffold material for SCI needs to have the following characteristics: good biocompatibility, favorable mechanical strength, proper degradation rate and suitable aperture. In detail, the scaffold should fill the lesion cavity, retain cells at the injury site, support the physical matrix, mediate directional growth and optimize the native microenvironment [45, 46]. Gelatin belongs to a macromolecule hydrophilic colloid. This biomaterial can easily bind to cells and growth factors for SCI [47]. Gelatin has been widely used in the treatment of SCI due to its low-cost, excellent biocompatibility, easy chemical modification, matrix metalloproteinase-mediated degradability and natural cell adhesion motif retention [48, 49]. Alginate is also a popular biomaterial used to fill injured spinal cord. In previous study, a silk fibroin/alginate scaffold with nerve growth factor (NGF) was transplanted into the site of transected spinal cord in rats [50]. The sustained release of NGF from the scaffold was conducive to the improvement of locomotor function and regeneration of neurons after 8 weeks. In this research, sodium alginate/gelatin hydrogels had appropriate mass volume ratio for 3D printing. The combination of the two biomaterials could make up for their shortcomings and exploit respective advantages. The results proved that the hydrogels prepared for 3D bioprinting had good biocompatibility, and the cells could be attached and survival in the scaffold. In this way, cells could play a better role after transplanted into the SCI model.

The composition and structure of the scaffold have a direct impact on its mechanical properties. 3D printing technology can be applied to print scaffolds that meet the requirements of surface morphology, shape and size of spinal cord implants. The porous structure of scaffolds could promote the transfer of oxygen, nutrition, and waste, as well as tissue integration and rapid vascularization, which has been reported in previous studies [13, 51–53]. The result of SEM indicated that the internal structure of hydrogels was porous, which conducive to the realization of the above functions. The ordered microstructure design of the scaffold was realized by 3D printing technology, which was conducive to guide axon regeneration. The results of rheological and mechanical tests showed that our hydrogels had good properties for printing and transplantation. In addition, the good compatibility of scaffold biomaterial was also an important factor to evaluate the treatment of SCI. We could observe a plenty of survived NSCs and OLGs in hydrogels at Day 5 after co-culture. These results indicated that 3D bioprinting sodium alginate/gelatin scaffold provided a good niche for adhesion and survival of NSCs and OLGs.

NSCs have been widely applied in the treatment of SCI animals. Several animal experiments have revealed that the transplanted NSCs could differentiate into new neural cells *in vivo*, provide nutritional support, compensate the loss of neurons, promote functional recovery and restore connectivity [25]. However, a great deal of studies showed that most of the transplanted NSCs would differentiate into astrocytes rather than neurons [24]. Moreover, the curative effect of NSCs alone was very limited, and it was far from achieving the goal of complete recovery of motor function. OLGs are a late evolutionary product to the cell bank of the vertebrate CNS. They are specially used to produce myelin sheath around axons to allow saltatory conduction of action potentials, to optimize information processing. After SCI, OLGs are particularly sensitive to the toxicity of the acute injury environment. The apoptosis of OLGs begins several hours after injury and continues for weeks. It is particularly harmful to the demyelination of neurons [54]. The lack of chronic, extensive demyelination is, at least partly, due to spontaneous remyelination of OLGs and Schwann cells after SCI [55]. Although OPCs proliferate and remyelinate spinal axons at the periphery of the lesion site, this process is limited because vast majority of OPCs undergo apoptosis before they differentiate into mature OLGs [56]. Interestingly, myelin becomes thinner and internodes become shorter than normal myelin when axons are remyelinated by OLGs [57]. As reported by Smith *et al*. [58], remyelination can recover axonal conduction velocity. As the main evidence, the research of Duncan laboratory supports the view that remyelination restores function [59]. Their research suggested that in myelin-deficient animals, the remyelination by transplanted OLGs could restored conduction velocity to a near normal level. Based these researches, we print the OLGs and NSCs together to improve the repair effect of SCI. H&E staining showed that there was no obvious cavity after transplant. Immunofluorescence results revealed that there were more MBP-, 5-HT- and NF-positive cells in the perilesional area of spinal cord treated with sodium alginate/gelatin scaffold loaded with NSCs and OLGs in comparison to scaffold and SCI groups. Axonal regeneration alone was not enough for the recovery of motor functions. In the process of regeneration, another key factor was remyelination of the regenerated axons [60]. The positive expression of MBP in the results (Fig. 5C and D) also showed that this 3D bioprinting scaffold combined with cells was helpful to promote remyelination and improve the therapeutic effect of SCI. Our findings clearly showed that the novel 3D bioprinting sodium alginate/gelatin scaffold combined with NSCs and OLGs is conducive to the regeneration of neuron and improvement of tissue structure at lesion site of SCI.

However, this study also has some limitations, there are still some deficiencies in the evaluation of neurological function, such as electrophysiological determination. To explore the fate of transplanted NSCs, we used the CM-Dil to label the NSCs which were applied to rat injured spinal cord in our 3D bioprinting scaffold system. At week 8 after transplantation, we failed to detect the fluorescence signal in sections of injured spinal cord samples (Supplementary Fig S1). Moreover, the result of immunofluorescence staining indicated that the neurons and astrocytes at the injured site were not directly derived from transplanted NSCs. Therefore, we supposed that the transplanted allogeneic NSCs ultimately did not survived after long-term transplantation and the more Tuj-1 positive cells within the lesion after 3D bioprinting scaffold/NSCs transplantation were from host axons, not from the direct differentiation of NSCs, perhaps by unknown indirect mechanism. Alternatively, transplanted NSCs worked by secreting neurotrophic factors [61]. We still have a lot of work to explore the functional recovery mechanism of the printing scaffold/NSCs *in vivo*.

## Conclusion

In summary, a biocompatible scaffold loaded with NSCs and OLGs was constructed via 3D bioprinting in this study. This scaffold was a good biomaterial for NSCs and OLGs loading, and exhibited therapeutic effect in the repair of SCI, by enhancing neural generation, axon growth, and locomotor functional recovery. We believed that this scaffold had the potential as clinical implant for the treatment of patients with SCI.

## Supplementary data

Supplementary data are available at *REGBIO* online.

## Funding

This work was supported by following grants: the National Key Research and Development Program of China (grant number 2017YFA0104304), the National Natural Science Foundation of China (grant numbers 81571213 and 82070459 to B.W., grant numbers 81800583 to Y.Y.X.), Key Project of Jiangsu Province (grant number BE2020765 to B.W.), Nanjing Medical Science and Technique Development Foundation (grant numbers QRX17006, QRX17057 and ZKX20016 to B.W.), Nanjing Medical Science and Technique Development Foundation (grant number YKK20071 to H.Y.), Jiangsu Provincial Plan for Mass Entrepreneurship and Innovation (2019, B.W.), and Project of Modern Hospital Management and Development Institute, Nanjing University/Aid project of Nanjing Drum Tower Hospital Health, Education & Research Foundation (grant number NDYG2020030 to B.W.).

*Conflicts of interest statement*. The authors declare that they have no known competing financial interests or personal relationships that could have appeared to influence the work reported in this paper.

## Supplementary Material

rbac038_Supplementary_Data
