# Supplementary material for: Three-dimensional bioprinting sodium alginate/gelatin scaffold combined with neural stem cells and oligodendrocytes markedly promoting nerve regeneration after spinal cord injury
Source: Regen Biomater. 2022 Jun 6;9:rbac038. doi: 10.1093/rb/rbac038 (PMC9255276; doi:10.1093/rb/rbac038)
Supplement: rbac038_Supplementary_Data [file rbac038_supplementary_data.docx]

**Supporting information**

**Three-dimensional b****ioprinting** **sodium** **alginate /** **gelatin scaffold** **combined with** **neural stem cells and oligodendrocytes markedly promoting nerve regeneration after spinal cord injury**

Shuo Liu^1,#^, Hui Yang^1,#^, Yuanyuan Xie^1^, Dong Chen^1^, ChenXu Tai^1^, Liudi Wang^1^, Peng Wang^2,*^, and Bin Wang^1, *^

1 Clinical Stem Cell Center, Nanjing Drum Tower Hospital, the Affiliated Hospital of Nanjing University Medical School, Nanjing, Jiangsu Province, China;

2 State Key Laboratory of Pharmaceutical Biotechnology, Department of Sports Medicine and Adult Reconstructive Surgery, Nanjing Drum Tower Hospital, the Affiliated Hospital of Nanjing University Medical School, Nanjing, Jiangsu Province, China;

^#^Both authors contributed equally to this work.

*Corresponding author (Bin Wang, Ph.D., 321 Zhongshan Road, Nanjing 210008, China. Telephone: 86-25-68182508; E-mail: [wangbin022800@126.com](mailto:wangbin022800@126.com), Peng Wang, 321 Zhongshan Road, Nanjing 210008, China. Telephone: 86-25-83106666; E-mail: 15850681759@163.com).

**Experimental section**

**1. Materials and Methods**

To explore the fate of transplanted NSCs, we used the living cell marker CM-Dil to label the NSCs. Neurospheres were digested into unicellular NSCs, and then labeled with CM-Dil (20 μg / mL) at 37 °C for 15 minutes and then 4 °C for 15 minutes. After centrifugation, the cells were washed with PBS and then incorporated into hydrogels. Subsequently, the NSCs-loaded hydrogels were shaped into fix-sized scaffolds according to the 3D printing system in the article. The operation method and transplantation process were the same as the article. Spinal cord tissues were sampled at different time points (week 1, 2, 4, and 8). Tissue samples were fixed, dehydrated, and sectioned for immunofluorescence staining by ant-Tuj-1, anti-GFAP, and DAPI. Images were captured under the Leica DMi8 confocal microscope.

**2. Results**

The results showed that the red fluorescence signal of CM-Dil could be observed in the first 4 weeks (Supplementary Figure 1). At week 8 after transplantation, we failed to detect the fluorescence signal in the sections of injured spinal cord samples. Moreover, the fluorescence signals of CM-Dil and Tuj-1 or GFAP do not coincide.

**
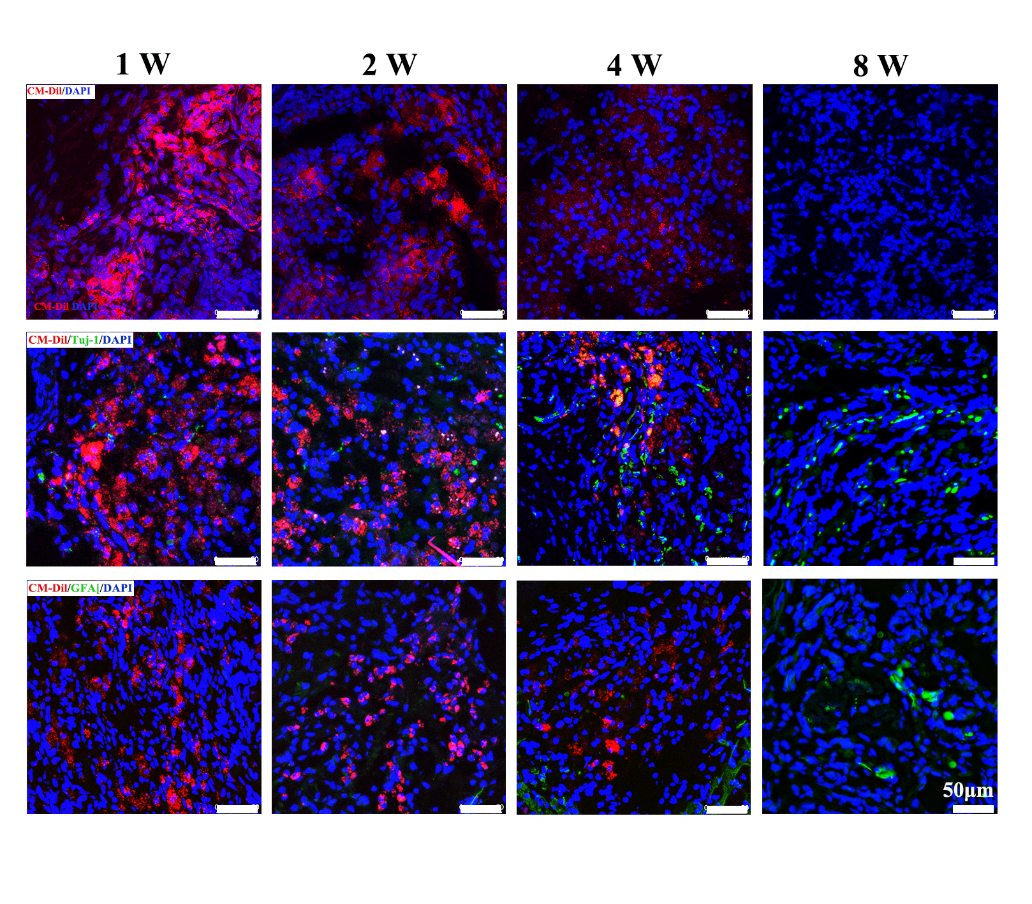
**

**Supplementary Figure 1 The survival of transplanted NSCs in 3D bioprinting scaffold *in vivo*.** The 3D bioprinting scaffold loaded with CM-Dil labeled NSCs and OLGs were transplanted to injured spinal cord, and the fluorescence signal of CM-Dil, Tuj-1, and GFAP were detected in the sections of spinal cord samples at week 1，2，4，and 8.
